# Neoadjuvant immunochemotherapy for resectable esophageal cancer: A study on efficacy and safety

**DOI:** 10.17305/bb.2025.11806

**Published:** 2025-04-01

**Authors:** Xiaomin Wang, Bingxu Li, Zhiyong Zheng, Weijie Wang

**Affiliations:** 1 Department 1st of Radiation Oncology, Anyang Tumor Hospital, Anyang City, Henan Province, China; 2 Department Thoracic Surgery, Anyang Tumor Hospital, Anyang City, Henan Province, China

**Keywords:** Immunotherapy, chemotherapy, neoadjuvant therapy, esophageal cancer, EC, neoadjuvant immunochemotherapy, nICT

## Abstract

The combination of immunosuppressants and chemotherapy has reshaped the treatment landscape for esophageal cancer (EC). This study aimed to evaluate the effectiveness and safety of a neoadjuvant immunochemotherapy (nICT) regimen in patients with resectable EC. A total of 99 eligible patients were included. Data on patient characteristics, nICT regimens, surgical approaches, postoperative outcomes, adverse events (AEs) related to neoadjuvant therapy and surgery, overall survival (OS), and disease-free survival (DFS) were collected. OS, DFS, and safety were the primary endpoints. Cox regression analysis was used to identify prognostic factors in the overall population. Additionally, exploratory research was conducted to assess the clinical value of blood immune indicators in predicting tumor regression. Following surgery, 99.0% of patients achieved complete resection (R0). After neoadjuvant therapy, the number of patients with stage T0N0 increased, with complete or moderate responses being the most common outcomes according to American Joint Committee on Cancer (AJCC)/College of American Pathologists (CAP)-tumor regression grading (TRG) evaluations (64.7%). The one-year OS and DFS rates were 91.6% and 49.3%, respectively. Grade ≥3 AEs related to neoadjuvant therapy occurred in 21.2% of patients, with gastrointestinal reactions being the most frequent (16 cases, 16.2%). No treatment-related deaths were reported. Grade ≥3 surgery-related AEs occurred in 10.1% of patients, with anastomotic leakage being the most common (six cases, 6.1%). Several factors were associated with significantly improved OS, including chemotherapy regimens combining paclitaxel with platinum, surgical approaches using laparoscopy or thoracotomy (left or right), an interval of ≤34 days between the last treatment and surgery, and the absence of positive lymph node detection. Higher cT staging was significantly associated with worse DFS. Blood immune markers, such as the neutrophil-to-lymphocyte ratio (NLR) and lymphocyte-to-monocyte ratio (LMR) were found to predict tumor regression in EC patients. In summary, nICT demonstrated favorable effectiveness and safety in resectable EC. The choice of platinum-based chemotherapy agents, rather than the type of immunosuppressant, was associated with prognosis. Moreover, a shorter interval (≤34 days) between the final nICT administration and surgery was linked to improved outcomes.

## Introduction

Esophageal cancer (EC) is one of the most common and aggressive malignant tumors worldwide, accounting for a significant proportion of cancer-related deaths. It ranks as the seventh most common cancer and the sixth leading cause of cancer mortality globally [1]. For resectable, locally advanced EC, direct surgery can be challenging due to high invasiveness or the presence of regional lymph node metastasis. In such cases, neoadjuvant therapy is commonly employed to improve patient survival outcomes [2, 3]. In recent years, a multimodal treatment approach—consisting of neoadjuvant chemoradiotherapy (nCRT) followed by surgery—has become the standard of care for resectable EC [4]. Compared to surgery alone, nCRT has been shown to improve both the R0 resection rate and overall survival (OS) in EC patients [5, 6]. However, this combined approach still has notable limitations. It does not significantly reduce the rates of local recurrence, distant metastasis, or severe treatment-related adverse events (TRAEs) [5]. Furthermore, the toxic side effects of concurrent chemoradiotherapy may considerably increase the risk of postoperative mortality, particularly in cases of esophageal squamous cell carcinoma (ESCC) [7, 8]. Therefore, there is an urgent need for a more effective and safer neoadjuvant therapy to improve long-term survival outcomes in patients with resectable EC.

In the era of immunotherapy, the emergence of immune checkpoint inhibitors (ICIs) has introduced a novel treatment approach for various tumors. Preclinical data suggest that neoadjuvant immunotherapy enhances the anti-tumor immune response by blocking immune checkpoint pathways in T cells, thereby exerting significant anti-tumor effects [9]. Its effectiveness and safety in treating EC have been confirmed in several clinical trials [10, 11]. Neoadjuvant immunochemotherapy (nICT) has gained popularity in recent years as a treatment regimen. Compared to the complexity and relatively high complication rate associated with nCRT, nICT is considered simpler and is associated with fewer complications [12, 13]. However, due to its relatively recent adoption, along with varying inclusion criteria and treatment strategies across studies, the impact of nICT on OS remains inconsistent, making its clinical applicability a subject of ongoing debate. To address these issues, we conducted a retrospective analysis of the prognosis and safety outcomes of EC patients who underwent surgery following nICT at our hospital. We examined clinical characteristics to identify risk factors influencing prognosis and investigated the potential of blood immune indicators to predict tumor regression. This study aims to provide a data-driven reference for developing neoadjuvant therapy regimens for resectable EC and to offer new insights into predictive markers of pathological response to neoadjuvant therapy.

## Materials and methods

### Patient samples

A total of 108 consecutive patients with resectable EC who received nICT at AnYang Tumor Hospital between August 2020 and September 2022 were included in this study. All patients underwent surgical resection following the completion of nICT. The study was conducted in accordance with the principles of the Declaration of Helsinki and received ethical approval from the hospital’s Institutional Review Board and Ethics Committee (2024KY10H01). Throughout the study, patients’ personal information and data were kept strictly confidential. As no sensitive content was involved, the requirement for written informed consent was waived.

**Figure 1.**
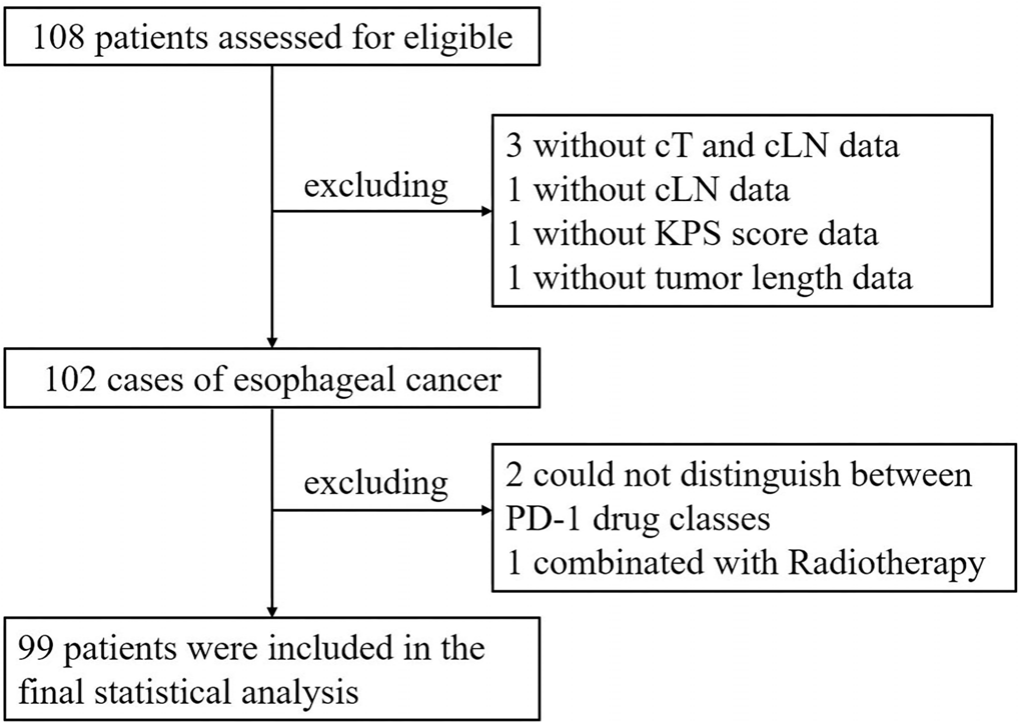
**Flowchart of the study cohort selection process.** KPS: Karnofsky Performance Status; PD-1: Programmed cell death-1.

To ensure the integrity of the sample information in this study, the inclusion criteria were as follows: (a) patients with a histologically confirmed diagnosis of EC; (b) no history of chemotherapy, radiotherapy, or immunotherapy for any cancer; (c) age over 18 years; (d) adequate organ function; and (e) availability of complete clinical data. The exclusion criteria included: (a) inability to identify the specific programmed cell death-1 (PD-1) inhibitor used; (b) receipt of other treatments, such as radiotherapy or targeted therapy, as part of combination therapy; and (c) presence of comorbidities, including esophageal perforation, autoimmune diseases, severe cardiovascular conditions, or other malignancies. A total of 99 eligible patients were included in the final analysis, with the detailed screening process illustrated in Figure 1.

As this was a retrospective study, a post hoc sample size analysis was performed based on OS. A one-sample rank test derived from the *t*-test was used, with parameters set for two-sided testing, a normal parent distribution, a correlation coefficient (ρ) of 0.3, and a significance level (α) of 0.05. The total sample size was 99. The analysis yielded a statistical power of 0.998, indicating excellent power.

### Procedures

All patients underwent clinical tumor assessment prior to treatment, including diagnostic biopsy, esophagography, endoscopic ultrasound, and computed tomography (CT) scans of the cervical, thoracic, and abdominal regions. Routine electrocardiograms and hematological tests were also performed.

Preoperative chemotherapy regimens primarily included platinum-based drugs (30–40 mg on days 1–3) combined with paclitaxel or albumin-bound paclitaxel (TP regimen, 300 mg on day one), platinum-based drugs with other agents, or paclitaxel monotherapy administered intravenously (IV). Immunotherapy drugs used were PD-1 inhibitors, including sintilimab (200 mg on day 0), camrelizumab (200 mg on day 0), toripalimab (240 mg on day 0), pembrolizumab (100–200 mg on day 0), penpulimab (200 mg on day 0), and tislelizumab (200 mg on day 0). Dosages were administered according to the respective drug instructions, and the treatment duration was adjusted based on the patient’s response and tolerance. Following completion of nICT, patients underwent esophagectomy with mediastinal lymph node dissection under general anesthesia. The specific surgical approach was determined by the patient’s condition, surgical judgment, and other relevant factors.

Throughout the treatment and follow-up period, all AEs were documented and graded for severity based on the NCI Common Terminology Criteria for Adverse Events (version 5.0) [14]. The follow-up period concluded in September 2023.

### Outcome measures

The primary endpoints were disease-free survival (DFS), OS, and safety. DFS was defined as the time from the start of neoadjuvant therapy to the first occurrence of recurrence, metastasis, or death from any cause. OS was defined as the time from the start of neoadjuvant therapy to the date of the last follow-up.

Postoperative tumor tissue was evaluated for pathological response to neoadjuvant immune checkpoint therapy (nICT) by two pathologists using the AJCC/CAP-TRG system, based on the extent of residual tumor cells. The AJCC/CAP-TRG system is defined as follows: Grade 0 (complete response): no viable cancer cells; Grade 1 (moderate response): single cells or small groups of cancer cells; Grade 2 (minimal response): residual cancer outgrown by fibrosis; Grade 3 (poor response): minimal or no tumor cell death, or extensive residual cancer [15].

### Ethical statement

This study was conducted in accordance with the principles of the Declaration of Helsinki and received ethical approval from the Institutional Review Board and Ethics Committee (2024KY10H01). The requirement for informed consent was waived by the committee due to the retrospective nature of the study.

### Statistical analysis

All statistical analyses were performed using SPSS version 26.0 and GraphPad Prism version 8.0. The normality of the data was assessed using the Shapiro–Wilk test. Continuous variables with a normal distribution are presented as mean ± standard deviation (SD), while non-normally distributed data are expressed as the interquartile range (IQR). Categorical variables are reported as frequencies and percentages (*n* [%]), and group comparisons were conducted using the chi-square test. Survival analysis was carried out using the log-rank test and illustrated with Kaplan–Meier (K–M) plots. Post-hoc pairwise comparisons were performed following the log-rank test, with Bonferroni correction applied to control for the type I error rate due to multiple comparisons. Univariate and multivariate Cox regression analyses were used to identify factors associated with patient outcomes following nICT. Additionally, receiver operating characteristic (ROC) analysis was conducted to evaluate the diagnostic performance of hematological immune markers in predicting pathological response to nICT, with the area under the ROC curve (AUC) used to assess predictive ability. A two-sided *P* value <0.05 was considered statistically significant.

## Results

### Baseline characteristics

This study included 99 patients with resectable EC treated between 2020 and 2022. The average age was 64.87 years, with a mean Karnofsky Performance Status (KPS) score of 86.72 and an average tumor length of 6.07 cm. The majority of patients were male (72.7%), and most tumors were located in the middle or lower esophagus (72.7%). Clinical T staging was predominantly T3 or T4 (90.9%), and lymph node involvement was observed in 91.9% of cases (Table 1). The median follow-up time was 19 months (range: 13–22 months).

**Table 1:** Patient baseline characteristics

**Characteristics**	**Value**
Age (Mean ± SD, years)	64.87 ± 7.37
*Sex (n, %)*	
Male	72 (72.7)
Female	27 (27.3)
KPS score (Mean ± SD, points)	86.72 ± 5.01
Tumor length (Mean ± SD, cm)	6.07 ± 1.94
*Tumor site (n, %)*	
Upper segment	27 (27.3)
Middle segment	42 (42.4)
Lower segment	30 (30.3)
*Clinical tumor stage (n, %)*	
T2	9 (9.1)
T3	70 (70.7)
T4	20 (20.2)
*Clinical nodal stage (n, %)*	
N0	8 (8.1)
N1	77 (77.8)
N2	14 (14.1)
N3	

SD: Standard deviation; KPS: Karnofsky Performance Status.

### Treatment information

The primary anti-PD-1 drug used in this study was sintilimab (75.8%), followed by camrelizumab (13.1%). Among the chemotherapy regimens, 93.9% of patients received a combination of paclitaxel and platinum, with albumin-bound paclitaxel being the predominant type (78.8%). In terms of platinum-based drugs, 99.0% of patients were treated with a platinum-containing regimen, primarily cisplatin (66.7%) and nedaplatin (27.3%). More than half of the patients (57.6%) underwent laparoscopic surgery, during which an average of 19.25 lymph nodes were removed. Additional clinical features are summarized in Table 2.

**Table 2:** Treatment information

**Characteristics**	**Value**
*Type of PD-1 inhibitor (n, %)*	
Sintilimab	75 (75.8)
Camrelizumab	13 (13.1)
Pembrolizumab	5 (5.1)
Penpulimab	3 (3.0)
Toripalimab	2 (2.0)
Tislelizumab	1 (1.0)
*Chemotherapy regimens-1 (n, %)*	
Paclitaxel + platinum	93 (93.9)
Others	6 (6.1)
*Type of paclitaxel in chemotherapy (n, %)*	
Paclitaxel	16 (16.2)
Albumin-bound paclitaxel	78 (78.8)
*Chemotherapy regimens-2 (n, %)*	
Platinum-based chemotherapy	98 (99.0)
Others	1 (1.0)
*Type of platinum drugs in chemotherapy (n, %)*	
Cisplatin	66 (66.7)
Nedaplatin	27 (27.3)
Oxaliplatin	3 (3.0)
Others	2 (2.0)
Course of chemotherapy (median [IQR], cycles)	2[2,2]
Number of preoperative immunizations (median [IQR], times)	2[2,2]
Time interval between last dose and surgery (Mean ± SD, days)	37.25 ± 10.61
*Surgical method (n, %)*	
Cavascope	57 (57.6)
Left thoracotomy	16 (16.2)
Right thoracotomy	22 (22.2)
Others	4 (4.0)
Number of lymph nodes removed (Mean ± S.D, count)	19.25 ± 7.97
Number of positive lymph nodes (median [IQR], points)	0[0,1]

IQR: Interquartile range; SD: Standard deviation; PD-1: Programmed cell death-1.

### Effectiveness

In this study, 99.0% of patients achieved complete resection (R0) following nICT. The proportion of patients at the T0N0 stage increased after nICT, with the AJCC/CAP-TRG assessment indicating either a complete or moderate response in 64.7% of cases, as shown in Table 3.

**Table 3:** Pathologic information after treatment

**Characteristics**	**Value**
*Surgical residuals (n, %)*	
R0	98 (99.0)
R1	1 (1.0)
*ypT (n, %)*	
T0	26 (26.3)
T1	22 (22.2)
T2	23 (23.2)
T3	25 (25.3)
T4	3 (3.0)
*ypN (n, %)*	
N0	63 (63.6)
N1	26 (26.3)
N2	8 (8.1)
N3	2 (2.0)
*CAP grading*	
Grade 0	26 (26.3)
Grade 1	38 (38.4)
Grade 2	17 (17.2)
Grade 3	18 (18.2)
*Induction therapy response assessment*	
TRG1	26 (26.3)
TRG2	38 (38.4)
TRG3	17 (17.2)
TRG4	18 (18.2)

TRG: Tumor regression grading.

**Figure 2.**
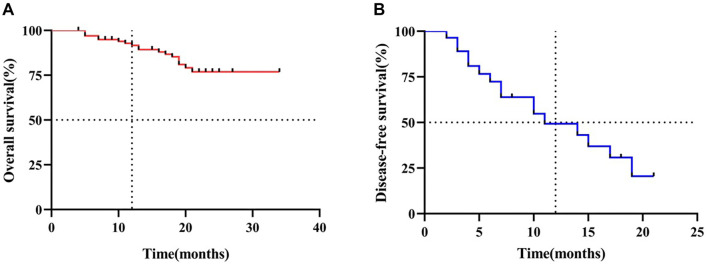
**K–M curves of OS (A) and DFS (B) for EC patients.** K–M: Kaplan–Meier; OS: Overall survival; DFS: Disease-free survival; EC: Esophageal cancer.

During the follow-up period, 18 patients (18.2%) died. Among these, 12 deaths (12.1%) were due to tumor progression, 1 (1.0%) was due to pneumonia combined with tumor progression, 3 (3.0%) were due to pneumonia, and 2 (2.0%) were attributed to unknown causes. The median OS for the total study population could not be determined, but the one-year OS rate was 91.6% (Figure 2A).

During the follow-up period, 28 patients (28.3%) experienced disease recurrence, metastasis after treatment, or death from any cause. Among them, 18 patients (18.2%) had tumor recurrence in the postoperative tumor region—15 in the mediastinal lymph nodes, three in the peritoneal lymph nodes, and one at the anastomotic site. Additionally, nine patients (9.1%) developed distant metastases after surgery, including three in the liver, two in the lungs, two in the bones, one in the brain, and one in the stomach. The median DFS was 11.0 months (95% CI: 4.0–18.0 months), with a one-year DFS rate of 49.3% (Figure 2B).

### Safety

Table 4 shows that 21.2% of all patients receiving nICT treatment experienced grade 3 or more severe AEs. The most common AE was gastrointestinal reactions, reported in 16 cases (16.2%), followed by bone marrow suppression in four cases (4.0%). Additionally, one patient (1.0%) experienced a grade 4 AE—dermatitis. However, throughout the entire neoadjuvant treatment period, there were no instances of dose reduction, treatment interruption, surgical delay, or death due to AEs.

**Table 4:** Neoadjuvant therapy-related AEs

**AE**	**Grade 3**	**Grade 4**
Digestive tract reaction	16 (16.2)	0
Bone marrow suppression	4 (4.0)	0
Dermatitis	0	1 (1.0)

Data are expressed as *n* (%). AE: Adverse event.

The incidence of surgery-related grade ≥3 AEs was 10.1%, with the most common being anastomotic leakage, reported in six cases (6.1%), as detailed in Table 5.

**Table 5:** Surgery-related AEs

**AE**	**All grades**	**Grade ≥3**
Anastomotic leak	15 (15.2)	6 (6.1)
Hemorrhage	2 (2.0)	0
Stress ulcers	1 (1.0)	1 (1.0)
Pleural effusion	1 (1.0)	1 (1.0)
Incisional fat liquefaction	1 (1.0)	1 (1.0)
Lung infection	1 (1.0)	1 (1.0)

Data are expressed as *n* (%). AE: Adverse event.

### Treatment subgroup analysis

Patients were grouped based on the type of PD-1 inhibitor received: the sintilimab group (*n* ═ 75) and the other drug group (*n* ═ 24). After treatment, no significant differences were observed between the two groups in terms of pathological information, OS, DFS, or AEs (OS: *P* ═ 0.143; DFS: *P* ═ 0.945) (Tables S1 and S2 and Figure S1). Patients were divided into two groups based on the chemotherapy drug received: the cisplatin group (*n* ═ 66) and the other drug group (*n* ═ 33). No significant differences were observed between the two groups in terms of pathological characteristics, OS, DFS, or AEs (OS: *P* ═ 0.505; DFS: *P* ═ 0.634) (Tables S3 and S4 and Figure S2).

### Exploration of prognostic factors

#### Overall survival (OS)

**Figure 3.**
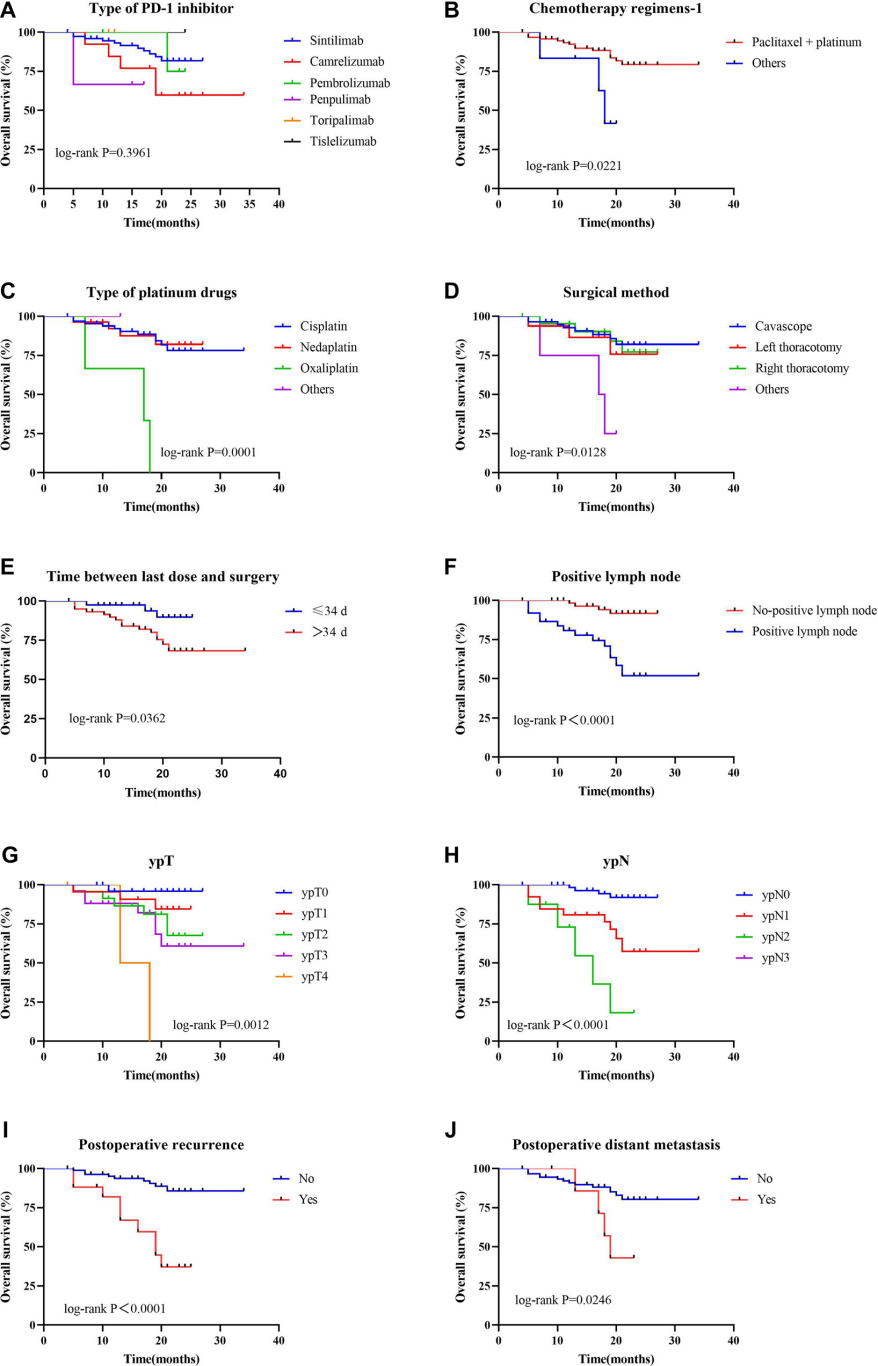
**K–M curves of factors related to OS in EC patients.** (A) PD-1 inhibitor type; (B) Chemotherapy regimen 1; (C) Platinum drug species; (D) Surgical method; (E) Last dose-to-surgery interval; (F) Positive lymph node; (G) ypT; (H) ypN; (I) Postoperative recurrence; (J) Postoperative distant metastasis. PD-1: Programmed cell death-1; K–M: Kaplan–Meier; OS: Overall survival; EC: Esophageal cancer.

Univariate analysis of OS showed that the type of platinum-based chemotherapy, positive lymph node status, ypT stage, ypN stage, AJCC/CAP-TRG assessment results, postoperative regional recurrence, and postoperative distant metastasis all had significant effects on OS (*P* < 0.05). Variables with a *P* value <0.1 in the univariate analysis were included in the multivariate analysis. The multivariate results identified the type of PD-1 inhibitor, surgical method, time interval between the last dose of medication and surgery, and postoperative regional recurrence as independent risk factors for OS (*P* < 0.05, Table S5). In addition, survival curves were plotted based on the univariate results. These showed that the type of PD-1 inhibitor had no significant effect on OS (*P* ═ 0.396, Figure 3A, Table S6). Patients who received a paclitaxel plus platinum chemotherapy regimen (*P* ═ 0.022, Figure 3B), underwent surgery via cavascope, left thoracotomy, or right thoracotomy (*P* ═ 0.013, Figure 3D, Table S6), had a last dose-to-surgery interval of ≤34 days (*P* ═ 0.036, Figure 3E), and had no positive lymph nodes (*P* < 0.0001, Figure 3F) demonstrated significantly better OS (*P* < 0.05). Furthermore, patients treated with oxaliplatin had significantly worse OS compared to those who received other platinum drugs (cisplatin or nedaplatin) (*P* ═ 0.0001, Figure 3C, Table S6).

#### Disease-free survival (DFS)

**Figure 4.**
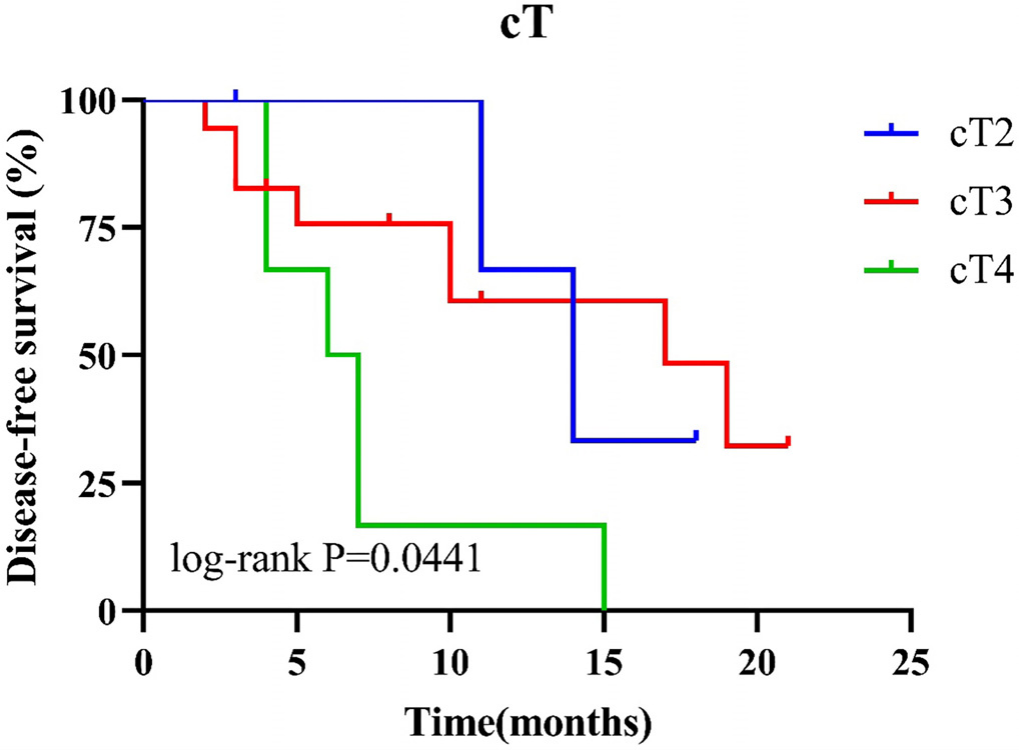
**K–M curve of cT staging and DFS in EC patients.** EC: Esophageal cancer; DFS: Disease-free survival; K–M: Kaplan–Meier.

Univariate and multivariate Cox regression analyses revealed no significant associations for any of the evaluated factors. However, K–M curve analysis indicated that cT staging was significantly associated with DFS, with higher cT stages correlating with poorer DFS outcomes (*P* ═ 0.044; Figure 4, Tables S6 and S7).

### Exploratory study

**Figure 5.**
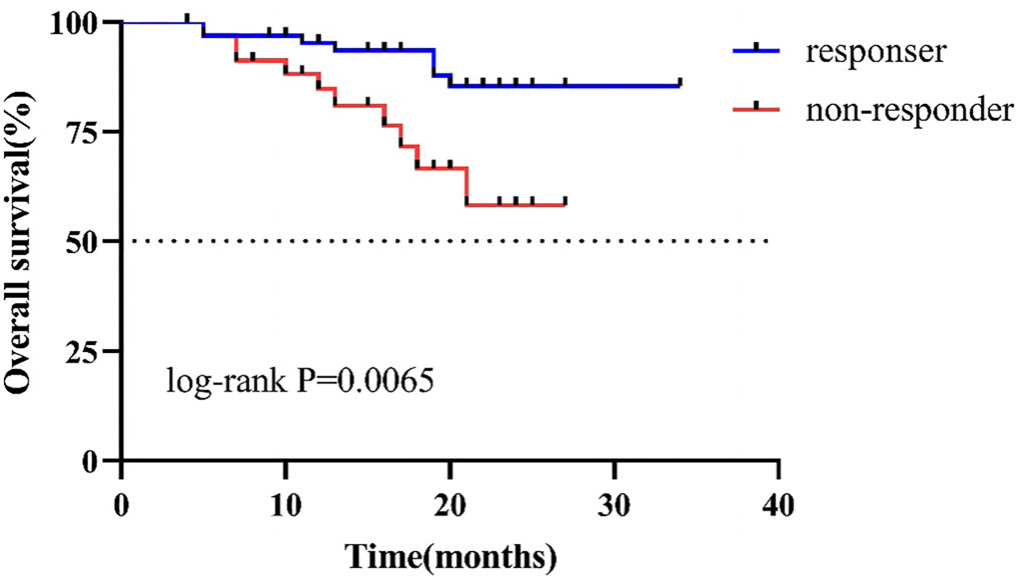
**K**–**M curves of responders and non-responders for OS.** K–M: Kaplan–Meier; OS: Overall survival.

We defined patients with CAP staging of 0–1 as responders (*n* ═ 64) and all other patients as non-responders (*n* ═ 35). K–M analysis revealed a significant difference in OS between the two groups, with responders demonstrating significantly better OS compared to non-responders (Figure 5).

**Figure 6.**
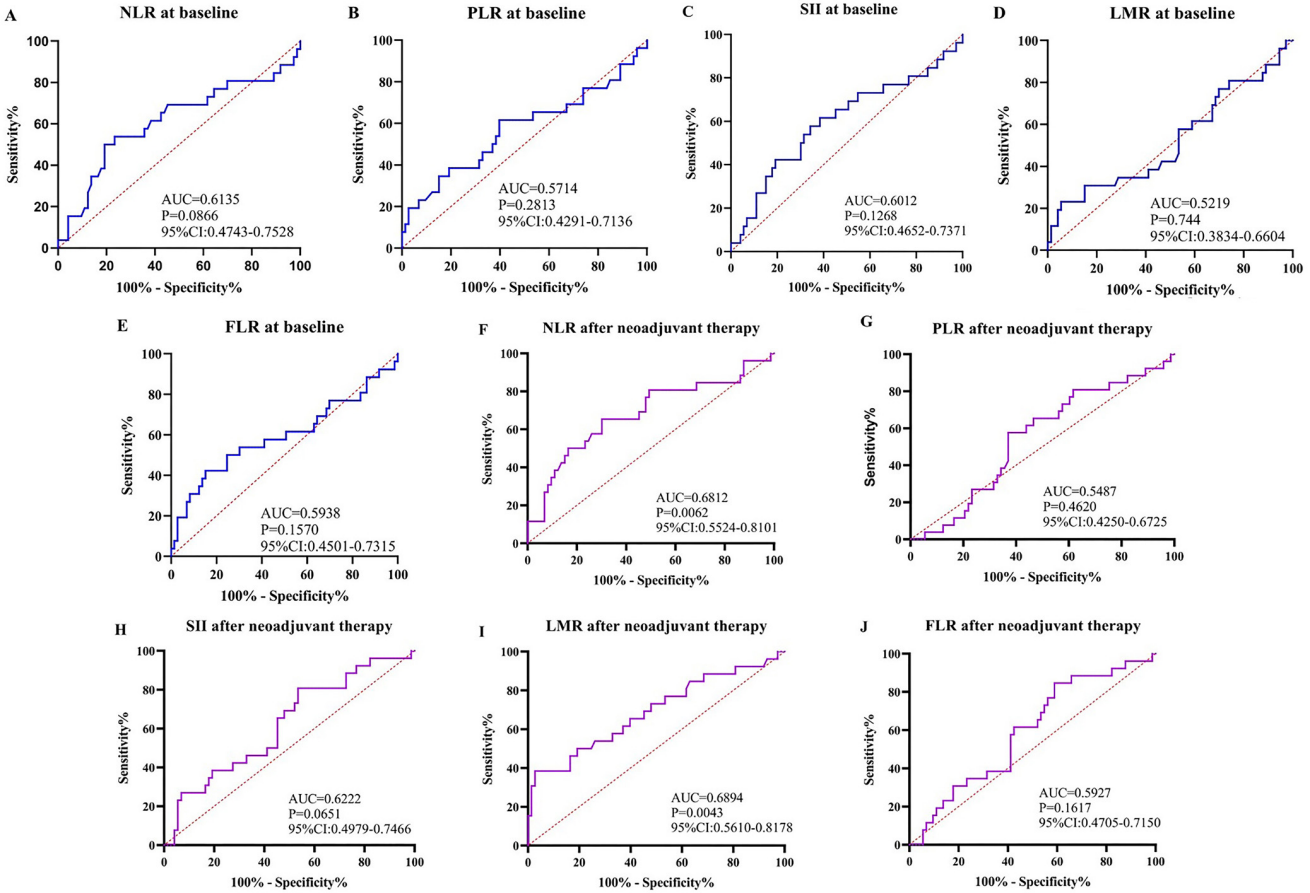
**ROC curve analysis of blood indicators for diagnosing EC.** (A–E) ROC curves of baseline NLR, PLR, SII, LMR, and FLR; (F–J) ROC curves of NLR, PLR, SII, LMR, and FLR after nICT. ROC: Receiver operating characteristic; NLR: Neutrophil-to-lymphocyte ratio; PLR: Platelet-to-lymphocyte ratio; SII: Systemic immune-inflammation index; LMR: Lymphocyte-to-monocyte ratio; FLR: Fibrinogen-to-lymphocyte ratio; EC: Esophageal cancer; AUC: Area under the ROC curve.

Since CAP staging was obtained through postoperative pathological examination and used to predict tumor tissue regression in advance, we chose to examine inflammatory markers in the blood—specifically the neutrophil-to-lymphocyte ratio (NLR), platelet-to-lymphocyte ratio (PLR), systemic immune-inflammation index (SII) (calculated as platelet count × neutrophil count/lymphocyte count), lymphocyte-to-monocyte ratio (LMR), and fibrinogen-to-lymphocyte ratio (FLR). After completion of neoadjuvant therapy, only NLR (with a cutoff value of 1.995, AUC ═ 0.6812, 95% CI: 0.5524–0.8101, *P* ═ 0.0062, Sensitivity ═ 0.6538, Specificity ═ 0.6938) and LMR (cutoff value 7.740, AUC ═ 0.6894, 95% CI: 0.5610–0.8178, *P* ═ 0.0043, Sensitivity ═ 0.3846, Specificity ═ 0.9726) were predictive of tumor regression. Other markers were not statistically significant (Figure 6). Although the predictive performance of these markers was not ideal, the findings suggest that investigating hematological immune parameters may offer a promising new direction for predicting pathological response in nICT, providing a basis for future research.

## Discussion

Surgical resection was long considered the preferred curative treatment for EC before the introduction of chemotherapy and radiotherapy. However, with ongoing advancements, neoadjuvant therapy combined with esophagectomy has become the standard treatment for patients with locally advanced EC. Following the successful application of ICIs in cancer therapy, immunotherapy has been incorporated into neoadjuvant regimens. Its efficacy and safety have been demonstrated in the treatment of advanced EC [16, 17]. nICT is now used as a first-line treatment for advanced EC [18], and it has been shown to significantly improve patient survival rates.

In this study, the average R0 resection rate following nICT treatment was 99%, slightly higher than in previous reports [5, 19]. For comparison, the NEOCRTEC5010 study reported a 98.4% R0 resection rate in the nCRT group [5], while Wang et al. [20] documented a rate of 97.3%. These findings suggest that nICT not only significantly improves the R0 resection rate but may also reduce the technical difficulty for surgeons in achieving complete resection of the primary tumor and lymph nodes.

In the CROSS trial, the median OS and DFS in the nCRT plus surgery group were 48.6 months and 37.7 months, respectively, compared to 24.0 months and 16.2 months in the surgery-alone group [21]. Similarly, the NEOCRTEC5010 trial demonstrated five-year OS and DFS rates of 59.9% and 63.6%, respectively, in the nCRT plus surgery group, vs 49.1% and 43.0% in the surgery-alone group [6]. However, some studies have found no significant OS improvement when comparing nCRT plus surgery to nCT plus surgery, although a trend toward reduced recurrence risk with nCRT has been observed [22]. In our study, the one-year OS rate for the overall cohort was 91.6%, while the one-year DFS rate was 49.3%. Due to the relatively short follow-up period, the median OS and DFS have not yet been determined. Continued follow-up will allow us to assess long-term (three-year and five-year) survival outcomes and evaluate whether nICT offers lasting survival benefits for patients with resectable EC.

In the safety analysis, the incidence of nICT-related grade ≥3 treatment-emergent AEs was 17.2%, indicating good tolerability and comparable to previous studies (16.3%) [19], with no AE-related deaths reported. The incidence of surgery-related grade ≥3 TRAEs was 10.1%, with anastomotic leakage being the most common complication, occurring in 6.1% of cases—lower than the 22% reported in the CROSS study [23]. This difference may be attributed to the surgical expertise of the clinical team. Overall, the safety profile of nICT is considered acceptable.

Subgroup analysis based on the type of PD-1 inhibitor revealed no significant differences in the effectiveness or safety contributions of various PD-1 inhibitors in neoadjuvant therapy. This finding aligns with results from a previous meta-analysis on neoadjuvant therapy for resectable EC, which indicated that ICIs (PD-1/PD-L1 inhibitors) do not introduce bias into clinical outcomes for EC patients [24]. Interestingly, however, subgroup analysis of chemotherapy agents showed that different chemotherapy drugs significantly affected OS in EC patients. Taxanes, platinum compounds, and fluoropyrimidines have long been regarded as key chemotherapeutic agents in the treatment of EC. Among patients receiving curative chemoradiotherapy, the combination of paclitaxel and platinum has been shown to provide better clinical responses and survival outcomes compared to fluoropyrimidine plus platinum regimens [25], a trend also observed in the ATTRACTION-3 trial [26]. Among platinum-based drugs, five types are commonly used in chemotherapy regimens [27]. Oxaliplatin, a third-generation platinum compound, offers several advantages, including broad-spectrum antitumor activity, efficacy against drug-resistant tumor strains, and relatively low toxicity. It has become a cornerstone of neoadjuvant therapy for EC [28, 29]. Recent studies suggest that chemotherapy regimens used as “immunotherapy induction therapy” may sensitize tumors to subsequent ICI treatment [30]. However, in our study, the small sample size of patients receiving oxaliplatin (*n* ═ 3) may have introduced statistical bias. Therefore, larger-scale prospective randomized controlled trials (RCTs) are necessary to determine whether this observed effect is specific to EC patients and to further clarify the comparative efficacy of oxaliplatin vs other platinum-based agents.

Esophagectomy is generally categorized into two types: open esophagectomy—typically performed via left or right thoracotomy—and minimally invasive esophagectomy, which has become more common in recent years [31]. Compared with traditional open surgery, minimally invasive procedures can reduce the risk of various complications and shorten hospital stays; however, they do not significantly lower patient mortality rates [32, 33]. For EC patients with metastasis or tumors in difficult-to-access locations, alternative treatment options are often considered based on the patient’s overall condition, as surgical resection may not be effective in such cases [34, 35]. Although this study found that patients who underwent laparoscopy, left thoracotomy, or right thoracotomy had better prognoses than those who chose other surgical methods, the choice of surgical approach depends heavily on factors, such as the patient’s physical condition, disease severity, and the surgeon’s expertise. Therefore, it is not possible to definitively determine a single “best” approach. Each case must be individually assessed to select the most appropriate surgical method.

In the era of multimodal therapy, surgeons often face challenges in determining the optimal timing for surgery. A patient’s condition and tumor response following neoadjuvant therapy can significantly influence surgical outcomes [36]. Delaying surgery may lead to the emergence of other uncontrollable complications. Therefore, selecting the appropriate timing for surgery is critical to optimizing patient outcomes. Currently, the recommended interval between the final cycle of nICT and esophagectomy—commonly referred to as time to surgery (TTS)—is largely based on protocols established for nCRT and neoadjuvant chemotherapy, with a traditional recommended window of 4–6 weeks [37]. However, a considerable number of patients undergo esophagectomy after a longer interval (> 6 weeks) for various reasons [37]. In clinical practice, there is no standardized or universally accepted TTS for patients receiving nICT. Most studies investigating the relationship between TTS and prognosis in EC patients focus on those receiving nCRT. Overall, while prolonging TTS may improve the pathological complete response (pCR) rate, it appears to be detrimental to long-term survival [38–40]. A recent meta-analysis supports this view [41]. Our findings are consistent with these studies: patients with a TTS of ≤34 days demonstrated better OS compared to those with a longer interval. We hypothesize that this may be due to a delayed immune activation effect induced by immune checkpoint inhibitors [42]. A shorter interval between the final dose of immunotherapy and surgery may allow the immune system to remain in an activated state, thereby enhancing anti-tumor responses and improving post-surgical OS. In contrast, a prolonged interval may result in the waning of peak immune activity, potentially altering the tumor microenvironment and enabling residual resistant tumor cells to evade immune surveillance—ultimately compromising survival. The optimal interval from neoadjuvant therapy to surgery likely varies between individuals, and at present, there are no definitive clinical indicators to guide timing after nICT. One study reported that timely surgery improved survival in patients without a clinical complete response, compared to delayed surgery [43]. This raises the question: can the evaluation of anti-tumor response (e.g., complete or partial remission) serve as a guide for determining surgical timing? More clinical trial data is needed to confirm the validity of this approach.

Our exploratory study on tumor regression after neoadjuvant immune checkpoint therapy (nICT) also provides valuable insights for identifying indicators that can help determine optimal surgical timing. Pathological remission—particularly pCR following neoadjuvant therapy—is closely associated with improved survival rates in patients with EC [44]. However, the CAP pathological grading system currently used in clinical practice is based on postoperative pathological evaluation, making it impossible to assess in real time for patient stratification and clinical decision making. Therefore, identifying effective biomarkers that can predict pathological response is critical for evaluating tumor regression. Immune indicators in peripheral blood and the tumor microenvironment significantly impact cancer prognosis [45, 46]. Various markers of systemic inflammation—including NLR, PLR, SII, LMR, and FLR—play indispensable roles in tumor development, angiogenesis, invasion, and metastasis. These markers often interact at multiple levels, collectively influencing survival and prognosis in cancer patients [47–49]. Take NLR and LMR as examples. Lymphocytes are key indicators of host immune status, contributing to anti-tumor immunity by recognizing tumor-associated antigens. Lymphocytopenia, in contrast, is associated with reduced survival in many cancers [50, 51]. Monocytes, which play a critical role in the tumor microenvironment, are considered indicators of tumor burden [52]. The LMR, which reflects both tumor-related inflammation and host immune status, has been linked to cancer-related mortality [53]. Similarly, NLR is recognized as an independent risk factor for poor OS and DFS in various cancers [54–56]. Neutrophils promote tumor progression by enhancing angiogenesis, damaging DNA, inhibiting T-cell-mediated anti-tumor responses, and facilitating metastasis [57]. Therefore, the combination of neutrophilia and lymphocytopenia—reflected in elevated NLR—may represent a shift toward a tumor-promoting microenvironment. A study assessing baseline immune levels found that both NLR and LMR can effectively distinguish responders from non-responders to nICT treatment [58]. Moreover, a meta-analysis indicated that clinical indicators, such as NLR and LMR have moderate prognostic predictive value [59]. These findings suggest that NLR and LMR may serve as predictors of pathological tumor regression after nICT. Although only these two markers have demonstrated a clear association with pathological response in EC patients, their predictive potential highlights the value of immune indicators in this context. Despite the current limitations in predictive performance, this line of inquiry guides us toward identifying new biomarkers—particularly immune-related factors in peripheral blood—with stronger predictive capabilities. We further hypothesize that, if a peripheral immune marker capable of accurately predicting pCR after nICT is identified, it may support a shorter TTS than is currently recommended. We plan to conduct further experiments to test this scientific hypothesis.

In this study, we found that cT was the only indicator significantly associated with DFS, with higher cT grades corresponding to worse DFS outcomes. EC is staged using the TNM system, which includes tumor infiltration depth (cT stage), lymphatic metastasis of malignant esophageal cells (cN stage), and distant metastasis (cM stage). Surgeons and oncologists use TNM staging to determine appropriate treatment strategies and timing. Accurate cT staging is critical for selecting the optimal treatment plan for patients with EC [60], and preoperative tumor volume has been identified as an independent prognostic factor for both PFS and OS in ESCC patients [61]. The depth of tumor infiltration is closely associated with the risk of lymph node metastasis [62], with higher cT grades indicating an increased risk. As a result, patients with deeper tumor infiltration are generally recommended to receive adjuvant therapy following surgical resection [63].

This study supports the use of nICT as a viable treatment option for resectable EC and explores the optimal interval between nICT and surgery. Our findings suggest that peripheral blood immune markers may serve as promising tools for predicting pathological response to neoadjuvant therapy, offering a potential strategy for evaluating the TTS. However, several limitations should be acknowledged. First, this is a retrospective analysis with a relatively small sample size, particularly among patients receiving immunotherapy. This limitation is more pronounced in subgroup analyses and the classification of adverse events, increasing the risk of statistical bias. Future studies should prioritize multicenter, prospective RCTs with larger sample sizes—especially across various patient subgroups—to enhance statistical power and reduce bias. Additionally, including data from diverse geographic regions in such multicenter studies would improve the generalizability of the findings. Second, due to the relatively short follow-up period, our study lacks long-term survival data. Ongoing follow-up is necessary to collect comprehensive information on long-term survival, recurrence, and late-stage adverse events, in order to better assess both the efficacy and safety of nICT. Future research should extend the follow-up period to provide a more thorough evaluation of these outcomes. Third, our study did not include data on the reasons for surgical delays, which may have introduced bias. Future investigations should incorporate detailed perioperative management data to better understand their potential impact on surgical timing and patient prognosis. Finally, while we assessed the predictive value of NLR and LMR separately, we did not construct a combined predictive model. Future research should explore multifactorial predictive models that integrate both biomarkers to enhance predictive accuracy and validate their clinical utility through multicenter studies.

## Conclusion

In conclusion, this study confirms that nICT holds significant clinical value in patients with resectable EC. It not only achieved a 99% R0 resection rate and a 91.6% one-year overall survival rate, but also demonstrated good safety. The study further revealed that the surgical interval and inflammatory markers may have a potential impact on prognosis, offering new insights into the development of personalized treatment strategies. Future large-scale prospective studies are needed to further validate these findings and optimize the comprehensive treatment plan for EC.

## Supplemental data

Supplemental data are available at the following link: https://www.bjbms.org/ojs/index.php/bjbms/article/view/11806/3806.

## Data Availability

The date and materials in the current study are available from the corresponding author on reasonable request.
